# Complete Genome Sequence of Campylobacter jejuni Strain G1, Isolated from a Patient with Guillain-Barré Syndrome

**DOI:** 10.1128/MRA.00505-21

**Published:** 2021-07-29

**Authors:** Andrey V. Karlyshev

**Affiliations:** a School of Life Sciences, Pharmacy and Chemistry, Faculty of Science, Engineering and Computing, Kingston University London, Kingston upon Thames, United Kingdom; University of Maryland School of Medicine

## Abstract

Here, I report the complete genome sequence of Campylobacter jejuni strain G1, belonging to Penner serotype HS1. One remarkable feature of the genome of this isolate is the presence of four copies of Mu-like prophages, of which none are present in some other strains, including the reference strain NCTC11168.

## ANNOUNCEMENT

Campylobacter jejuni strain G1, isolated from a Guillain-Barré syndrome patient (1), has the smallest known capsular polysaccharide locus (2–6). A draft genome sequence of this strain (7) allowed the identification of genes responsible for the biosynthesis of a highly efficient multidrug efflux pump involved in multidrug resistance (8). In this study, the complete genome sequence of this strain was derived using a hybrid approach (9).

The strain was grown on brain heart infusion agar (Oxoid, USA) for 48 hours at 37°C in a microaerobic atmosphere. Approximately 10^8^ CFU were resuspended in 120 μl of Tris-EDTA (TE) buffer with lysozyme (0.1 mg/ml) and RNase A (0.1 mg/ml), incubated for 25 min at 37°C, followed by addition of proteinase K and SDS to 0.1 mg/ml and 0.5%, respectively, and incubated for 5 min at 65°C. Genomic DNA was purified using an equal volume of solid phase reversible immobilization (SPRI) beads (Beckman, USA) and resuspended in elution buffer (Qiagen, Germany).

A sequencing DNA library was prepared using the Nextera XT library prep kit (Illumina, San Diego, CA) following the manufacturer’s protocol. Assembly of 2 × 250,566 paired short reads (average, 228 bp) produced by the Illumina NovaSeq 6000 platform using SPAdes software v.3.7 resulted in 25 contigs (1 to 406 kb); the *N*_50_ value was 120,957 bp. Long read genomic DNA libraries were prepared with the Oxford Nanopore Technologies (ONT; United Kingdom) SQK-RBK004 kit and/or SQK-LSK109 kit with the native barcoding EXP-NBD104/114 (ONT) kit with 500 ng of DNA. Barcoded samples were pooled into a single sequencing library and loaded in a FLO-MIN106 (R.9.4.1) flow cell in a GridION system (ONT). Reads were adapter trimmed using Trimmomatic 0.30 with a sliding window quality cutoff of Q15 (10). Genome assembly was performed using Unicycler v.0.4.9b (11). Default parameters were used for all software unless otherwise specified.

The contigs were joined by using the GridION Oxford Nanopore system, which produced 6,413 reads up to 161 kb with an *N*_50_ value of 40,557 bp. The size of the complete genome sequence was 1,778,460 bp with GC content of 30.43% and 153.96× coverage. It was annotated by the NCBI Prokaryotic Genome Annotation Pipeline (PGAP) v.5.1 (12) using the best-placed reference protein set and GeneMarkS-2+, which identified 1,894 genes, including 1,783 protein-coding genes, 55 pseudogenes, 44 tRNAs, 3 sets of rRNAs (5S, 16S, and 23S), and 3 noncoding RNAs (ncRNAs).

**FIG 1:**
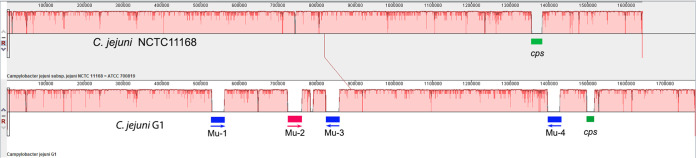
Comparison of complete genomes of C. jejuni NCTC11168 and G1 strains using Mauve alignment tool v.2.4.0 (13). Gaps in the genome of strain G1 indicate three almost identical Mu-like prophages (in blue) and a more distantly related Mu-like prophage (in red). Green gaps in both genomes denote capsular polysaccharide loci having very low sequence similarity.

A comparison with the draft genome assembly using Mauve software v.2.4.0 (13) revealed misassembled contigs, despite that they were verified by read mapping, confirming previously reported issues when using read mapping for assessment of draft genome assemblies (14). The draft assembly was 56,930 bp smaller than the complete version. The additional sequences in the complete version were identified by the Mauve tool (13) revealing long repeats represented by four Mu-like prophages. The sequences of three prophages were almost identical (32 kb) and shared extensive similarities with the fourth prophage (37 kb). Mainly due to the presence of these prophages, the genome of G1 was found to be 137 kb larger than that of the reference strain NCTC11168 (15), with the remaining parts of the genomes present in a colinear arrangement (Fig. 1).

The study highlights problems with shotgun draft genome sequencing potentially leading to misassemblies and data misinterpretation and contributes to a better understanding of the evolution of this important pathogen and its lifestyle.

### Data availability.

This whole-genome shotgun project has been deposited at DDBJ/ENA/GenBank under the accession number CP073712, BioProject number PRJNA261878, and BioSample number SAMN03077634. The version described in this paper is the first version, CP073712.1. Raw sequences were deposited under SRA accession numbers SRR14425908 (long reads) and SRR14425515 (short reads).
